# When Suddenly Nothing Works Anymore Within a Team – Causes of Collective Sport Team Collapse

**DOI:** 10.3389/fpsyg.2018.02115

**Published:** 2018-11-06

**Authors:** V. Vanessa Wergin, Zsuzsanna Zimanyi, Christopher Mesagno, Jürgen Beckmann

**Affiliations:** ^1^Chair of Sport Psychology, Department of Sport and Health Sciences, Technical University of Munich, Munich, Germany; ^2^School of Health and Life Sciences, Federation University Australia, Ballarat, VIC, Australia; ^3^School of Human Movement and Nutrition Sciences, University of Queensland, Brisbane, QLD, Australia

**Keywords:** collective team collapse, negative momentum, emotional contagion, performance contagion, key player collapse

## Abstract

Collective team collapse occurs when multiple players of a sport team experience a sudden and extreme underperformance within a game. To date, minimal research has been conducted on the causes of collective team collapse. Thus, goals of this study were to explore perceived causes of collective team collapse in different sports and to define team collapse in contrast to negative momentum. To investigate factors causing and maintaining collective sport team collapse, an inductive, exploratory qualitative analysis of individual interviews was conducted. Semi-structured interviews were carried out with 10 athletes of professional German teams of various sports playing in between first and fourth division. Participants were interviewed about a team collapse event they had experienced with their team during the past year. Data were collected and analyzed using a grounded theory methodology. Collective team collapse appeared to be induced by a temporal cascade of causes rather than by single triggers. This cascade included antecedents, which represent factors that make the occurrence of a team collapse more likely; critical events, which include specific events within the game that trigger a team collapse; as well as affective, cognitive, and behavioral outcomes that foster a maintenance of the collapse. Within this theoretical framework, social factors, such as decreased performance contagion or emotional contagion, played crucial roles in causing a team collapse. These results illustrate that collective team collapse is more than the sum of individual choking of multiple players at the same time. In conclusion, a new definition, differentiating team collapse from negative momentum, is introduced. Furthermore, a process model of causes of collective team collapse is proposed. The results provide first insights into causes of collective collapse in a variety of team sports. The developed model is supposed to help future research to better connect to practice and to support athletes, coaches, and sport psychologists.

## Introduction

Most team sport athletes are familiar with the following phenomenon: Their team’s game is going well and suddenly, from one moment to the other, performance drops so considerably that nothing seems to work anymore for the team. The 2017 Super Bowl describes one of the most significant examples of such a sport team collapse in recent years: The Atlanta Falcons led 28-3 against the New England Patriots during the second half and had a 98.9% statistically calculated chance to win the game, but unexpectedly lost 28-34 in overtime (Rafferty, 2017). The Falcons’ performance was described as one of the toughest losses in Super Bowl history, where one could observe a team falling apart. Such an incident is an example of collective team collapse, which occurs when a team is in the lead and abruptly loses control over the game (Boss and Kleinert, 2015). It describes situations where unexpectedly nothing seems to be working anymore within a team’s performance capability. Apitzsch (2006) defined team collapse as occurring “when a majority of the players in a team sport suddenly perform below expected level in a match of great, often decisive, importance in spite of a normal or good start of the match or when a team underperforms right from the start of a match” (p. 38).

Adler and Adler (1978) were among the first researchers to describe the sudden, extreme shifts in sport team performance with the term “momentum”. These changes in physical performance can be both positive and negative, where positive momentum is when everything seems to work well in a team, and negative momentum is when the team underperforms collectively (Den Hartigh et al., 2014). Momentum can shift from positive to negative as well as from one team to the other during a game (e.g., Cotterill, 2012; Den Hartigh et al., 2014). Recent research in the area of momentum in sport showed that negative psychological changes, such as collective efficacy and team cohesion, as well as interpersonal coordination seem to play a role in causing negative momentum (Den Hartigh et al., 2014). Furthermore, Boss and Kleinert (2015) discovered the social effect of performance contagion as an underlying mechanism of negative momentum with rowing pairs. These results are difficult to transfer to actual sport teams though, since they focused on teams consisting of two members, randomly assigned to each other. Group processes within teams consisting of more than two members, who have been part of a team for months or years, may be different and more complex. Various models of momentum have been developed so far. Taylor and Demick (1994) proposed the Multidimensional Model of Momentum, consisting of a momentum chain that explains the development of both positive and negative momentum. This momentum chain includes precipitating events, followed by changes in cognition, affect, and physiology, which cause behavioral changes that lead to an increase or decrease in performance. This change in performance combined with changes in cognition, physiology, affect, and behavior of the opponent team then causes a change in outcome.

Momentum constitutes a phenomenon that is closely related to collective team collapse (Cotterill, 2012), but existing research has failed to sufficiently distinguish between the two phenomena. Apitzsch (2009), the first known researcher to investigate collective team collapse, used Taylor and Demick’s (1994) momentum chain and extended it through qualitative data from players of a handball team that had just recently experienced collective team collapse. Apitzsch (2009) explains that two different causal chains, happening at two different points in time within a game, could evoke what he labels as collective collapse. The first chain, prior to the start of a match, includes either negative thoughts, leading to negative emotion and resulting in a passive playing style, or positive emotions, leading to positive thoughts and resulting in overconfidence and mistakes during the game. The second chain, occurring at the end of a game, involves a critical event within the game that leads to negative cognitions, which then cause passive behavior and subsequent negative performance. Apitzsch (2009) developed these causal chains through a qualitative case study exploring the causes of team collapse with a sport team. He found inappropriate behavior, failure of the role system, negative communication, opponent’s change in tactics, and scoring by the opponent to be major causes of the handball team’s collective collapse but misses to specify in detail what these factors stand for. Although Apitzsch’s (2009) case study provides some insight into team collapse, knowledge of factors causing team collapse across different teams and game situations is limited. In order to identify factors that make the occurrence of team collapse across various teams and team sports more likely, qualitative research with different situations and various types of sport is needed (e.g., Crust and Nesti, 2006; Apitzsch, 2009).

Several factors influencing momentum (Taylor and Demick, 1994) and possibly collective team collapse (Apitzsch, 2009) as well are related to psychological processes and the perception of momentum, which are often referred to as “psychological momentum” (Iso-Ahola and Mobily, 1980; Crust and Nesti, 2006; Gernigon et al., 2010; Mortimer and Burt, 2014). Iso-Ahola and Mobily (1980) defined psychological momentum as “an added or gained psychological power, which changes interpersonal perceptions and influences an individual’s mental and physical performance” (p. 391). Congruent to momentum, psychological momentum can be either positive or negative and Taylor and Demick (1994) argue that positive or negative shifts in performance (momentum) can only happen if they are recognized by the team (psychological momentum). The existence of psychological momentum has been discussed broadly in the literature (e.g., Taylor and Demick, 1994; Crust and Nesti, 2006; Mortimer and Burt, 2014). Cornelius et al. (1997), for example, argue that attributions of positive or negative psychological momentum are no more than mental labeling processes to evaluate or describe performance. In their model, negative psychological momentum is an outcome rather than a cause of bad performance. Vallerand et al. (1988) introduced the Antecedents–Consequences Model (ACM) of psychological momentum, suggesting that the impact of positive psychological momentum on performance depends on personal factors, such as motivation, as well as on situational factors, such as audience behavior. While the ACM distinguishes antecedents from consequences of psychological momentum, it focuses on positive psychological momentum only and does not consider negative psychological momentum, which is problematic (Apitzsch, 2009).

While empirical research on causes of momentum, psychological momentum, and collective team collapse is still developing, many studies have investigated the causes of individual performance failure (i.e., choking under pressure or simply “choking”). Choking can be defined as “performance decrements under circumstances that increase the importance of good or improved performance” (Baumeister, 1984). Various theories exist about causes of choking, which are related to concentration difficulties, including distraction (Wine, 1971), a high self-focus (Baumeister, 1984; Beilock and Carr, 2001), and shifts of attentional focus (Eysenck et al., 2007; Oudejans et al., 2011). In order to bridge the gap between individual and team sport performance failures, Hill and Shaw (2013) conducted a qualitative study examining individual choking in various types of team sports. By interviewing team sport athletes, Hill and Shaw (2013) found important games, expectations, individual responsibility, presence of an audience, and physical as well as mental errors to be important antecedents for the occurrence of individual choking within a team sport. Anxiety, distraction, and perceived control were mechanisms triggering choking within a team. Furthermore, moderators, such as team cohesion or motivational climate, as well as perceived consequences, such as a significant drop in performance or negative affect, were crucial for the occurrence of individual choking in teams. These findings offer initial insights into factors influencing individual choking in a team environment that could be similar to causes of the choking of a whole team. It, however, does not consider the reasons of a collective decrease in a team’s performance.

While individual choking is well explored already, research related to the collective collapse of an entire team is still limited. It further appears that existing research applies two different terms, namely collective team collapse, and negative momentum, to describe this phenomenon without differentiating between the terms. Momentum is even used to describe both the individual and collective underperformance of athletes. Thus, the current qualitative study does not only aim to investigate athletes’ perception of causes of collective sport team collapse, but also attempts to define team collapse in contrast to the seemingly similar construct of negative momentum. In order to overcome limitations of Apitzsch’s (2009) case study, a sample of athletes from different teams in a variety of sports was included, which we expect to result in a more global view of causes of team collapse.

## Materials and Methods

### Philosophical and Methodological Orientation

One purpose of the study was to gain an understanding of athletes’ lived experiences and perceptions of collective team collapse in their specific team sport environment, and a second to develop an inductive theory that displays the phenomenon in these specific situations. Considering this purpose and the limited empirical research exploring the causes of team collapse, a constructivist–interpretivist grounded theory methodology (Charmaz, 2006) was used to collect and analyze data and subsequently derive a substantive theory (Holt, 2016; Weed, 2017). Constructivists reject the idea of objectivity and assume that the view of the world and certain phenomena rely on individual perceptions of it (Weed, 2017). Since these individual perceptions are subjective due to personal experiences, it is impossible to gain a perfect, unbiased view of the world. Thus, a grounded theory methodology can be used to understand multiple biased perspectives of reality in order to achieve a view that is plausible and representative of the data. Charmaz (2006) described grounded theory as “a systematic, yet flexible methodology for collecting and analyzing qualitative data to construct theories that are grounded in the data themselves” (p. 2). Consequently, she proposed that it was both a method and product of inquiry (Sparkes and Smith, 2014). This methodology enables “an analytical interpretation of the participants’ worlds and the processes constituting how these worlds are constructed” (Charmaz, 2005, p. 508) to generate theory from data.

### Participants

Participants consisted of 10 athletes (seven male, three female) from different teams within various sports (i.e., four volleyball, three basketball, two soccer, and one field hockey). Athletes’ ages ranged from 19 to 30 years (*M* = 24.30, *SD* = 3.74). Participants’ criterion for inclusion was 10 years or more of experience in the sport and varied between 10 and 20 years (*M* = 15.60, *SD* = 3.57). The time they were a member of their current team varied between 0.5 and 14 years (*M* = 4.95, *SD* = 4.49). All participants were playing in between first and fourth division in Germany and therefore either on a national or the highest regional level. The experience level of the athletes was required to ensure that a lack of skills and abilities was not the reason for the experienced team collapse.

### Interview Guide

We developed a semi-structured interview guide to investigate athletes’ perceptions of causes of collective sport team collapse. The first author, with a background in psychology, and the second author, with a background in sport science, designed potential interview questions dealing with underlying factors of team collapse and with processes occurring within the team during the collapse event. During 4 × 4-h sessions, the first and second authors discussed the questions with the third author, who is experienced in qualitative research. The three authors then discussed the third author’s critique and review until consensus on a final version of the interview guide was reached.

The first section of the interview guide consisted of a short colloquial description of team collapse to ensure participants were envisioning the same phenomenon. It was developed during 2 × 4-h sessions by the first, second, and third authors. The description included several aspects mentioned to be important for team collapse in existing literature (Apitzsch, 2006; Boss and Kleinert, 2015), namely that the team’s performance decreased more than usual, that this happened unexpectedly and that nothing seemed to be working anymore within the team during the collapse. Participants then described a similar experience with their team preferably within the last 12 months or within the last 5 years at maximum to capitalize on memory recall. Even though we provided a lengthy timeframe (up to 5 years), all participants were able to recall a team collapse event within the last year. The second section (Questions 1–7) consisted of questions about details of the team collapse, such as, at what point during the game it took place or how many players were involved. The content of the third section (Questions 8–12) included questions about the impact the team collapse had on players and game. A sample question for this section was “To what extent did the team collapse influence the further course of play?” The last question (Question 13) specifically asked about influencing factors of collective team collapse and was posed last in order not to affect participants’ answers to previous questions. To conclude the interview, participants were asked whether there was anything else they found to be relevant regarding the topic of collective team collapse (Question 14). The full interview guide is included in the Supplementary Data Sheet 1.

### Data Collection

Given the purpose of the study, two different sampling methods were used to recruit participants. The purposeful sampling method of criterion-based sampling was chosen as the principal sampling method to approach athletes of various German sport teams. In order to fulfill the sampling criteria, participants needed to: (a) be members of a team sport consisting of more than two players, (b) play in between first and fourth division, (c) have experience in playing the sport for 10 years or more, (d) have experienced a team collapse event with their current team, and (e) be willing to talk about the team collapse event. Athletes who fulfilled these criteria were recruited for the study and the purpose of the research was explained upon arrival for the interview. After implementation of the initial interviews, theoretical sampling was applied to recruit further participants. First results showed that key players and their performance seemed to play a crucial role in the team collapse process and that athletes attributed more responsibility to key players, who fulfill a leadership role in the team. Although there are several formal and informal leadership roles present within teams, and even though team captains do not necessarily fulfill a principal informal leadership role in their team, it is undeniable that captains hold a formal leadership role. Therefore, in order to include the perception of causes of team collapse of players with a leadership role in the team, team captains were predominantly recruited during theoretical sampling. The retrospective semi-structured interviews were carried out by seven different interviewers, whereby the first author interviewed three athletes and the second author interviewed one athlete. The additional five trained assistant interviewers were involved in interviewing due to organizational issues, such as the implementation of interviews in different parts of Germany at the same time. Furthermore, it was important for the interviewers to be knowledgeable in the sport to fully engage in the interview. All interviewers were trained professionally on semi-structured interview conduction for 12 h prior to carrying out any interviews. To further ensure consistency among the different interviewers, they tested the interview guide with two athletes each as a pilot testing phase, while being supervised by the first and second authors. Specific feedback on the interview conduction was provided before interviewers completed actual interviews with study participants. This procedure was repeated twice and occurring differences in the application of the interview guide were discussed between interviewers, and first and second author until consensus about the interviewing process was reached.

The duration of the interviews ranged from 20 to 40 min (*M* = 31.14; *SD* = 6.97). Interviews were carried out in German, which was the native language of all participants and interviewers. All participants were informed that participation was voluntary and that goals of the study were to identify underlying factors and mechanisms of the phenomenon of team collapse. They were assured the right to withdraw from the interview at any time without penalty. Participants were further informed that audio records would be used for research purposes only and that recorded data would be treated confidentially. Additionally, they were asked to sign a declaration of consent, stating that they had been informed about the purpose of the study and agreed with audiotaping of the interview. All participants gave written informed consent in accordance with the Declaration of Helsinki. The study did not involve any invasive or potentially dangerous methods and therefore, in accordance with the German Research Foundation (DFG) and in accordance with the guidelines of the Department of Sport and Health Sciences at the Technical University of Munich, did not require a formal ethics approval.

### Data Analysis and Trustworthiness

All interviews were audio recorded and manually transcribed verbatim, resulting in 146 pages of single-spaced text. The first two authors analyzed the interview transcripts following Charmaz (2006) suggestions on an inductive thematic grounded theory analytical procedure. Both authors read the interview transcripts multiple times to enhance familiarity with the content. During initial coding, interview data were analyzed using incident-to-incident coding and *in vivo* codes (Charmaz, 2006). An example for initial coding is given on the basis of the following quote: “Passes don’t arrive anymore, throws don’t reach the basket, you don’t achieve stops, talking of defensive stops, and everything becomes difficult and then you think. Then you get this panic and this panic doesn’t help at all and so the collapse continues.” While the first part of this quote was coded as unforced error and bad defense, the second part was coded as anxiety. During focused coding (Charmaz, 2006), concepts generated through open coding were reassembled into categories and subcategories to better explain the perceived causes of team collapse. The categories unforced error and bad defense for example were classified as subcategories in the superior category of unforced error accumulation. Theoretical integration included the investigation of relationships between categories and sub-categories in order to gain an understanding of the connections between categories. In the presented example, unforced error accumulation was linked to player anxiety. Throughout the process of initial and focused coding, constant comparison was applied, continuously comparing new data with already developed concepts, categories, and relationships between categories. Simultaneously, theoretical memos were used during initial and focused coding and contained various interpretations and relations between the identified categories in the form of graphical mind maps. Memos were further used to guide integration of categories into a theoretical framework in seeking to plausibly explain the relationships between causes of team collapse. Data saturation was identified when interviews did not provide any new information for the development of further categories. Theoretical saturation occurred when fresh interview data did not reveal any new properties of the established categories of the theoretical process model (Charmaz, 2006). Data saturation and theoretical saturation were reached after eight interviews, which is why we stopped interviewing after 10 participants.

Data collection and analysis followed the suggestions of Smith and McGannon (2018) for conducting rigorous qualitative research. Therefore, methods used in the past to demonstrate methodological rigor, such as member checking, inter-rater reliability, and the notion of universal criteria, have been omitted in the analytical process as they were “shown to be ineffective for verification, trustworthiness, or reliability purposes” (Smith and McGannon, 2018, p. 1). In order to support the analytical process and enhance methodological rigor, assumptions made by researchers were regularly compared to newly gathered data (Weed, 2017). In addition, graphical illustrations of the developing model were used during data analysis to help researchers think theoretically instead of descriptively (Holt, 2016). The third author, who was not involved in data analysis initially, acted as a “critical friend” (Sparkes and Smith, 2014; Smith and McGannon, 2018) and challenged the abstracted qualitative categories as well as the theoretical model developed by first and second author. In this role, he provided feedback on the classification of categories from an independent, external expert perspective. Categories and the developed theoretical model were then discussed extensively between first, second, and third author. The resulting process model was furthermore evaluated *post hoc* using “fit, work, relevance, and modifiability” as quality criteria (Weed, 2017, p. 153). It was evaluated to fit the complex phenomenon of team collapse as experienced by the participants and to work through explaining the relationship between various causes of team collapse. The phenomenon was also judged to be relevant for teams competing in a league system and the developed model was found to be suitable for future adaptions.

## Results and Discussion

**FIGURE 1:**
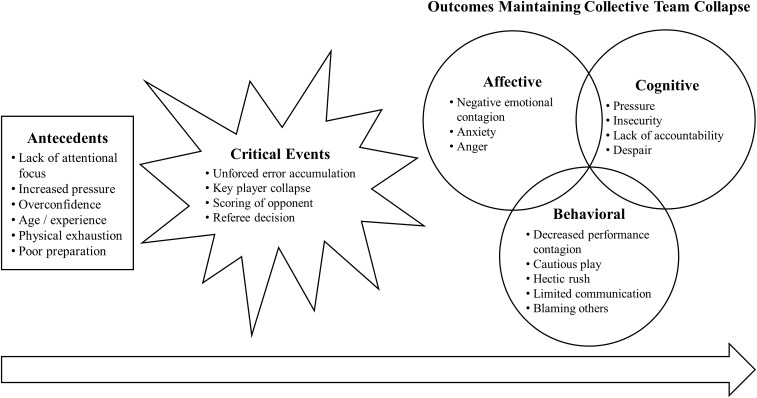
Process model of causes of collective sport team collapse. Figure 1 is a two-column fitting image.

To gain a better understanding of causes of collective team collapse, we analyzed athletes’ perceptions of the phenomenon and possible causes of it. We included interview responses from all 10 participants in the results. The interview analysis showed that athletes described collective team collapse as being evoked and maintained by a cascade of causes rather than by specific single triggers. Participants found these causes to be in a temporal order, which is why the results of the interview analysis are also presented in a sequential order. Some factors seemed to be present before the underperformance of the team and tended to make the team prone to a team collapse. These factors were labeled as antecedents. Other factors seemed to describe a specific event within the game triggering the actual team collapse, which were classified as critical events. Furthermore, participants named factors that seemed to be a result of the critical event, leading to the further maintenance of the team collapse. These factors maintaining the collapse were further divided into affective, cognitive, and behavioral outcomes. Figure 1 shows a schematic illustration of the relationship between these factors in the form of a process model. The process model of collective sport team collapse collapse illustrates the results of our analysis and our interpretation of the data, but not necessarily the characteristics of the phenomenon of collective team collapse. With the depiction of our results, we chose a linear representation to account for a general trend in the athletes’ narration, implying a temporal difference between antecedents and outcomes of team collapse. A process model represented this data best since it incorporates the temporal differences between facilitators of collective team collapse reported by athletes. As indicated by group dynamics research, however, group phenomena are often subject to cyclical and dynamic rather than linear processes. While this seems to be contradictory to our representation, we want to emphasize that dynamic processes may still play a major role within our temporal, linear framework, but were not subject of our investigation in this study. Future research needs to further explore the specific processes within the phenomenon of collective team collapse.

### Definition of Collective Team Collapse

Based on our study as well as existing literature in the field, we define collective team collapse as a sudden, collective, and extreme underperformance of a team within a competition, which is triggered by a critical situation that interferes with the team’s interplay, a loss of control of the game, and ultimately the inability of the team to regain their previous performance level within the game.

Collective team collapse was described to happen suddenly, since athletes for example said “suddenly our scoring stops” or “you had a buffer of 10 points and then suddenly it’s plus minus two”. The collapse was further mentioned to be collective, meaning that all players in the team were involved. Athletes described that it started with several individual players but that “at the end all are involved” and that collective team collapse is “generally a team thing”. Athletes also stated that the collapse situation evoked an extreme underperformance, where “nothing worked anymore” for them. All participants further emphasized that the collective team collapses they had experienced were induced by a specific “key moment,” a critical event on the court like “a harmless duel,” a “very harsh duel,” or a situation where “the referee decided against the team.” These critical events were described to create a game situation where “nothing fit together anymore” in the team and the team “couldn’t play together anymore” or “didn’t get back into the normal game rhythm,” because they were playing “unstructured,” with “no clear scheme.” This was captured in the definition as “a critical situation that interferes with the team’s interplay.” Athletes perceived that the collapse also went along with a loss of control of the game situation, because they felt that they “couldn’t make anything work anymore” and that “control had been taken out of their hands.” Moreover, participants described that they were “trying to get out of the collapse” but this seemed “to make the collapse even worse.” This was summarized as the inability of the team to regain their previous performance level. At the end of the definition, we added that the collapse had to happen within a game in order to differentiate it from underperformances of teams during several games.

### Antecedents

Participants described that prior to the start of a match certain conditions had an impact on the occurrence of a team collapse event, which likely increased the team’s vulnerability to a team collapse. One condition reported by athletes was a *lack of attentional focus*. Participants described attention within the team to be very low prior to the occurrence of a team collapse. They explained that teammates were either distracted or did not focus on the game anymore. Athlete 1 (basketball) described that his teammates were mentally absent and how this lack of concentration transferred to other players: “I have the feeling that we went on the court and it was like we missed the start of the game. Maybe one or two [players] were there a little, but the rest was ‘sleeping’ and you infected the others with it.” While Athlete 1 reported an absence of concentration at the beginning of the match, other athletes described that their perception of the opponent as weak was what caused a lapse in concentration. For example, Athlete 2 (volleyball) described that the attentional focus of her team was minimal because everything went smoothly and was possibly too easy for them: “A team collapse happens if everything seems to work on its own, if the opponent makes mistakes and the concentration decreases. You have to keep concentration up, no matter if you score or not. If everything goes too smooth, you lose focus. You have to continue playing as precisely as possible, as if you had to give everything.” This preexisting lack of concentration seems to increase the likelihood of unforced errors and therefore team collapse occurrence, which is a well-known cause of individual choking (e.g., Eysenck et al., 2007; Oudejans et al., 2011; Fryer et al., 2017). Similarly, athletes seemed to perceive a collective absence of minds that may have transferred between team members as a precursor of collective team collapse. Morgan et al. (2013) could, similarly, show that resetting a team’s focus could alleviate pressure and increase a team’s performance.

Another factor mentioned to be present prior to a team collapse was *increased pressure*, which was likely due to perceived importance of the game, presence of a huge audience, or an audience of significant others. Athlete 10 (basketball) said: “There was this pressure on us. It was the last game of the season and you wanna win the title. And the audience that was there and the atmosphere and you know it’s the last game. The whole season, the practice, what you invested. This is the final game … and I believe that performance wouldn’t have decreased so significantly in another game. There was so much external pressure in this game … and I’m someone who then becomes relatively anxious.” This finding is in line with Martens et al. (1990) model of competitive anxiety that explains the experience of pressure through perceived importance of the game. Marchant et al. (1998) showed that perceived importance of an event also plays a key role in increasing competitive anxiety in golfers. It appears that athletes experienced pressure through the importance of the game, which seemed to cause an increased fear of losing, and thereby may have fostered the team’s vulnerability to experience collective collapse.

Participants also perceived *overconfidence* to be important prior to a team collapse. Athletes described that their team had already perceived a “win” in their heads, because they had a big lead or because the opponent was not playing well, before the team collapse occurred. Athlete 9 (soccer) stated: “We were in the lead 3-0 at half time and we were quite euphoric. The atmosphere was – it was a really good day, nothing could go wrong anymore. Then we even scored the 4-0 after half time. Actually, there wasn’t much that could happen anymore. And then the first goal of the opponent was okay but from the second on there was a jolt going through the team. Because you don’t wanna ruin it.” It seems that the team was very confident about winning the game and thus surprised when the opponent scored, whereby their confidence of winning shifted to a fear of losing the game. Athlete 5 (soccer) even described his team being arrogant due to their ranking and dominance in the game, which likely led to mistakes of the players due to their careless style of playing: “I would say it [the reason for the collapse] was extreme arrogance on our side. We were ranked first in the league and we thought we would easily win … It [the collapse] happens when you feel overly secure and you try difficult passes, play extremely careless and thoughtless, and make mistakes.” Apitzsch (2009) and Hill and Shaw (2013), similarly, found overconfidence to be an antecedent of team collapse and of individual choking in teams, respectively. It seems that overconfidence increases the chances of failure (Baumeister et al., 1993; Apitzsch, 2009) by causing an overestimation of own abilities, leading to a more reckless and careless behavior. This behavior appears to increase the chances of failure and to make a team more vulnerable to the occurrence of team collapse.

Participants also found the composition of *age and experience* of the players on the court to be important before a team collapse event. Athletes suggested that a larger number of younger players on the court with less experience in difficult game situations increased the likelihood of the team to experience a collapse. For example, Athlete 6 (volleyball) explained the cause of the team collapse as follows: “The team was set up newly with many younger players, who hadn’t experienced such a situation before. You could see that they were, well not actually desperate, but they didn’t know how to handle the situation at all and then everything became hectic …And the coach started substituting players way too late”. The athlete argues that younger and unexperienced players were more affected by team collapse situations than older players were. This is similar to what Apitzsch (2009) describes as a lack of experience causing team collapse. Repeated experience of stressful situations was found to foster resilience to stress (Fletcher and Sakar, 2012) over time. It seems like athletes learn from their experiences with team collapse situations and are less susceptible to other team collapse situations afterward. This resilience could also be facilitated by a knowledgeable other like the team’s coach.

Players also indicated *physical exhaustion* to be an antecedent for team collapse. Athlete 10 (basketball) explained that his team was exhausted from a previous game and therefore not able to show its regular performance in the team collapse game: “Maybe [the collapse happened] because we were a little tired. Because we had semifinals the day before, where we played really well and then we were a little tired the next day …And then there was this extreme collapse.” Fatigue has been found to cause individual choking (Hill and Shaw, 2013) as well as negative momentum in teams (Taylor and Demick, 1994). If the whole team is fatigued from semifinals on the previous day, their exhaustion may cause a lower level of play or more mistakes within the team and thereby make a collapse more likely.

Furthermore, participants reported *poor preparation* of the team to be important prior to a team collapse. This poor preparation included an insufficient warm up, as Athlete 1 (basketball) explains: “I think it [the collapse] came from half-time, when we didn’t warm up enough. You have a break of 10–15 min and after talking to others and relaxing a little, you should focus during the break and warm up for 8–10 min to prepare, to get back in the game, but we didn’t do that, were not focused, and couldn’t play well.” In addition, insufficient practice before the game seemed to increase the risk of a team collapse, as Athlete 8 (field hockey) described: “I believe 2 weeks before, the basis for this event was founded because we didn’t practice and the effort and intensity of practice decreased, which is why we couldn’t follow the speed of the opponent during the first minutes.” Within choking research, Hill and Shaw (2013) reported that poor physical preparation caused fatigue and individual choking. Complementary to this, Morgan et al. (2013) found that thorough preparation fosters team resilience in difficult match situations.

### Critical Events

All participants described a specific trigger, an event or situation within the game that caused their team’s actual collapse. A very common factor describing such a critical team collapse event was *unforced error accumulation*, meaning that several individual players produced errors within the game at the same time or in succession. For example, Athlete 6 (volleyball) stated: “After the first set was finished, nothing worked anymore at all in the second set. I believe we lost that set by 12 or 13 and this was not related to any individual person, it was the whole team. The individual players started to make mistakes they didn’t make before, meaning serving, mainly during attacking, many balls just hit the block and then there was this pressure and tension in the team that didn’t vanish during the whole game anymore.” The accumulation of individual errors is likely a key factor that may lead to an underperformance of the whole team and to perceived pressure and tension. Athlete 8 (field hockey) expanded on this statement. The athlete proposed that several unforced individual mistakes led to a collapse of the tactical approach of the team and therefore to the actual collapse: “The collapse happened early in the game, where both teams were still scanning each other and several simple mistakes happened, which the opponent took advantage of. It was relatively simple individual mistakes of single players that eventually undermined the whole play system of the team and therefore made it very easy for the opponent to score.” The inability to maintain the play system due to the mistakes of the players appears to allow the opponent to score and cause the team to fall behind. Mistakes have been shown to play a crucial role in relation to a team’s underperformance but tend to be classified as a symptom or outcome of team collapse (Apitzsch, 2009), negative psychological momentum (Jones and Harwood, 2008), or negative momentum (Taylor and Demick, 1994) rather than as a trigger. A collapse of a team’s play system due to errors is a new finding to team collapse literature.

Some players further described that the poor performance of key players had a particular impact on the team and that *key player collapse* was what evoked a loss of points and underperformance of other players. Athlete 1 (basketball) stated: “I have the feeling that it was two or three [players] at the same time, but important players, who reacted too slowly and then there was a bad pass caused by two, but somehow the third, who could get the ball, didn’t do anything either.” Athlete 7 (volleyball) further explained: “We got a key player, who is very stable in reception, and she suddenly couldn’t manage anything anymore and that continued within the team …That was what caught my eye, that nothing worked anymore for her and shortly after that the same thing happened to the other players.” Somehow, the team seems to be strongly oriented toward key players and their underperformance appears to immediately cause an underperformance in other players. Apitzsch (2009), similarly, found that key players underperform and fail to do what is expected of them, which leads to an underperformance of the whole team. The underperformance of a key player may cause a decrease in perceived self-efficacy (Bandura, 1977) of the teammates resulting in a collective underperformance of the team.

Further to errors within their team, several players described *scoring of the opponent* to have a crucial impact on performance of their own team, triggering the team collapse. Athlete 3 (basketball) stated: “We were in the lead with three points in the first quarter and then in the second quarter we lost 9-33. We really did score only nine points. It started when the opponent scored all their throws and nothing worked anymore for us, we lost the ball, produced turnovers.” The opposing team scoring points during this time seemed to disturb their team’s course of play. This happened even if the own team was playing well before, as Athlete 2 (volleyball) explained: “So we have a run, meaning everything works, the interplay and everything; and then there’s this momentum when the opponent surprisingly scores and then we lost five points in a row because we couldn’t play the ball anymore.” Scoring of one team might lead to negative momentum for the other team demonstrating a momentum shift for both teams (Taylor and Demick, 1994). Apitzsch (2009) explained that factors associated with the opponent, such as scoring, have a moderating effect on the strength of the team collapse.

Athletes also reported that a perceived wrong *referee decision* was a critical team collapse event. Athlete 5 (soccer) described that the team complaining about the referee’s decision was what caused the team collapse: “There was this harmless duel in the middle [of the playing field]; we knew it was a foul, but we complained and stopped and they continued playing and were running up to our goal. They didn’t score but they gained back hope and played euphorically and induced a lot of pressure.” Jones and Harwood (2008) have found that poor refereeing decisions have a possible impact on psychological momentum, but did not specify in what ways. In this study, it seems that the interruption, the refusal of the team to continue playing, and the negative emotion about the perceived wrong referee decision had a negative impact on team performance and a positive impact on the mindset and performance of the opponent. Apparently, players perceived the referee’s decision and the game situation resulting from it as unfair and felt angry and helpless about it, which seemed to cause a drop in team performance.

### Outcomes Maintaining Team Collapse

All athletes further described that the critical team collapse event seemed to have changed certain processes within their team that led to aggravation and maintenance of the team collapse. These changes included emotional, cognitive, and behavioral factors and appeared to be mutually dependent upon each other. Several of the athletes’ statements reported especially emotional and cognitive outcomes to be so interrelated that it was impossible to identify a causal relationship in their perception (e.g., insecurity and anxiety). Factors were identified as emotional factors, if they represented one of the six basic emotions (Ekman, 1989), which can be identified from an individual’s facial expressions, and as cognitive factors if they incorporated cognitive evaluations of the situation. Some of these cognitive outcomes seem to operate through emotional reactions, which is why they are presented as inter-related dimensions in Figure 1.

#### Affective Outcomes

Affective outcomes were emotional changes that athletes described due to the critical team collapse event that fostered the maintenance of the collapse. Emotional contagion is defined as the transfer of emotion and moods within a group (Barsade, 2002). Players reported *negative emotional contagion* within their team due to the collapse situation. Athlete 7 (volleyball) described how the mood of her team changed during the collapse: “When we lost the first few points, everything was okay, we were like ‘okay, we’re gonna do this!’ Because in volleyball, it happens that you lose two or three points. But we lost more and more points and even if we scored in between, it [our mood] became more negative on the field and no one wanted to take the ball anymore.” Player 6 (volleyball) explained how negative emotions increased within the team and led to despair: “Some players became aggressive, others went quiet. I believe that emotions play a crucial role in volleyball and we didn’t lift ourselves up on our own points anymore and desperation became bigger and bigger, like a vortex.” Many studies have reported an association between negative emotions and individual underperformance (e.g., Hill et al., 2009; Barsade and Gibson, 2012; Hill and Shaw, 2013) as well as underperformance of a sport team (Kelly and Barsade, 2001; McEwan and Beauchamp, 2014). Similarly, collective positive emotions have been shown to be positively related to team resilience and team performance (Morgan et al., 2017). Furthermore, researchers have found a link between mood of the whole team and individual players (George, 1990; Totterdell, 2000) resulting in the assumption of an emotional contagion effect within a team. Apitzsch (2009) also reported emotional contagion as an outcome of the handball team’s collapse he investigated. Using Taylor and Demick’s (1994) theory and Apitzsch’s (2009) extension, it seems that negative affect and negative emotional contagion may have an impact on athletes’ cognition and evoke negative thoughts that, besides the underperformance itself, may maintain the collapse.

Athletes also described that *anxiety* was what maintained the collapse within their team and was crucial to the team collapse. Athlete 10 (basketball) explained: “We were 2-7 behind and then we received this run against us and then you start to panic, and that panic doesn’t help at all and that’s how the collapse remains. I believe if we would have been more relaxed and would have kept cool, we may have been able to manage the collapse.” Athletes also specified that they experienced a fear of making mistakes, which led to cautious play and caused even more errors. Athlete 6 (volleyball) described it as follows: “The quality of our play got worse in every possible way. We knew we could do better but no one performed anymore, especially in attacking, many balls were hit into the block blindly, serves weren’t hard anymore, because you had fear of failure. It’s hard to say but this also went along with a more hectic way of playing.” Especially fear of losing the game, fear of negative evaluation, and panic about the collapse itself seemed to be causes of a hectic way of playing and thus of a remaining underperformance of the team. This supports findings reported by Apitzsch (2009) as well as in choking under pressure literature on how anxiety associated with failure leads to decreased performance (Hill et al., 2009; Otten, 2009; Mesagno et al., 2012). Mesagno et al. (2012) use self-presentation theory (Schlenker and Leary, 1982) to explain that anxiety in individuals increases, when they want to impress others but do not believe in their own success. By applying self-presentation theory to this study, pressure and the desire to show good performance may be antecedents of the team collapse event, which increase even more through the critical event that seems to bring along a lack of self-efficacy. The resulting anxiety may, in turn, prohibit effective processing of task-relevant information and lead to maintenance of choking (Hill et al., 2009), or collective team collapse.

Participants described *anger* as another affective outcome. They explained how they themselves or other players within their team expressed their anger on the court due to their dissatisfaction with their team’s or their own performance. Athlete 10 (basketball) said: “Suddenly nothing works anymore and people get angry and yell at each other because of a mistake.” Athlete 9 (soccer) described how mistakes of teammates can lead to anger in individual players: “If your neighbor starts doing weird things, it has an indirect impact on you, too. Because it annoys you and makes you angry and then you are not as focused as you should be during the next action …and if a player causes four or five failures in a row, the team becomes uneasy. It can happen that two start to yell at each other and then the next yells at them.” This increasing anger in the team seems to prevent the team from finding their way back to a regular performance, possibly, as Athlete 9 describes, because they are busy dealing with their emotions and cannot focus on their actions or the game anymore. One participant in Apitzsch’s (2009) study also described the emotion of anger in relation to the team collapse. Other studies, similarly, found increased frustration in athletes due to their own choking (Hill and Shaw, 2013).

#### Cognitive Outcomes

Cognitive outcomes were perceptions, thoughts, and thought processes about the team collapse that maintained the team’s underperformance. Participants perceived that *pressure* resulting from the team collapse event hindered them from returning to an effective play. Athlete 7 (volleyball) described how pressure developed from the feeling of falling behind and the necessity to score in order to end the collapse: “During the game, nervousness increases because you think ‘Okay, you HAVE [emphasis added] to score now’. And that blocks the head even MORE because it’s always about HAVING to do something.” It seems that, as an outcome of the perceived pressure, the player experienced her thought processes as being blocked. Pressure being an outcome of the team collapse event itself besides being an antecedent present before the occurrence of team collapse has not been reported in other studies so far. It is assumed though, that underperformance caused by individuals or several team members, further increases pressure, which then again maintains the team collapse as kind of a vicious cycle.

The critical team collapse event as well as the pressure resulting from it often seemed to cause perceived *insecurity* in players. Some athletes explained that this was what caused them making even more mistakes. Athlete 9 (soccer) stated: “Then there’s insecurity, I’d say. If things don’t work and you don’t have a good day, you don’t trust in yourself and do more things that you normally wouldn’t do.” Athlete 7 (volleyball) further described how insecurity spread within the team during a team collapse and how it increased even more, if the key player did not perform well: “If you’re insecure on the court already, you look at the key player and if she plays good you think: it’ll work out somehow. And if she collapses you think: Okay, if she can’t do it, how am I supposed to?” The choking of a key player in this case seems to further increase insecurity in other players, which made them underperform as well. Increased insecurity caused by a team collapse event has also been reported by Apitzsch (2009) and is similar to low self-confidence and a perceived lack of ability as reported by Jones and Harwood (2008) during the experience of negative psychological momentum.

This perception of insecurity was closely related to the perception that the team suffered from a *lack of accountability* of the individual players. Athlete 5 (soccer) described how, especially in difficult situations, responsibility was handed over from one player to another: “You realized that this is a team sport and you can easily shift responsibility to the next one, meaning players are passing and then they hide a little.” Athlete 7 (volleyball) explained that this was also related to insecurity and anxiety associated with potential failure, meaning that players handed responsibility to their teammates so they themselves would not be responsible for the next mistake: “Everyone thought ‘oh god I don’t wanna take the ball anymore, you take it, you do the mistake.” This shift in responsibility has not been reported in team collapse literature so far. Morgan et al. (2013, 2015), however, show that collective accountability is an important factor for a team’s resilience, especially when the team experiences setbacks. The effect of diffusion of responsibility in collapsing teams may be explained by the social loafing process in groups (Karau and Williams, 1993) to some extent. Another explanation, given by Athlete 8, might be that anxiety about making mistakes is what leads to avoidance behavior in the athlete and therefore to the transfer of responsibility. Atkinson and Feather (1966) explained how fear of failure causes avoidance of situations in which the threat of failure is present or in which ability is being evaluated.

Furthermore, athletes stated to have experienced increased *despair* within the team during the collapse. Athlete 6 (volleyball) described that helplessness and despair within his team increased like a vortex due to the experience of team collapse: “Some players became aggressive, others went quiet. I believe that emotions play a crucial role in volleyball and we didn’t lift ourselves up on our own points anymore and desperation became bigger and bigger, like a vortex …You could sense a certain helplessness; no one knew why that happened or what actually happened in that situation.” Athlete 6 describes how the despair and helplessness perceived by his team originated from negative emotions on the court. It seems that emotions became so negative that the team felt desperate and hopeless about the game, did not know what was going on anymore, and ultimately resigned since they did not believe to be able to change anything anymore. Jones and Harwood (2008), similarly, reported hopelessness as an outcome of the experience of negative psychological momentum. Murayama and Sekiya (2015) observed resignation and despair to play an important role in individual choking under pressure, as well. Hill and Shaw (2013) even reported that athletes would withdraw from a game and demand to be substituted due to their own underperformance.

#### Behavioral Outcomes

Behavioral outcomes were behaviors that athletes showed as a reaction to the team collapse event. Participants reported *decreased performance contagion* to be occurring within their team, meaning that decreased performance of individual players transferred to other players of the team. Athlete 8 (field hockey), for example, described how the bad performance of individual players caused other players of the team to fail as well: “The collapse was triggered by individual mistakes and that led to collective failure. I would say that the individual mistakes caused an insecurity in the whole team and mistakes increased even more.” This contagion effect seems to be especially strong, if the player making initial mistakes is a key player. Athlete 7 (volleyball) specified that the key player “infected” the team with her bad performance and caused collective collapse: “We have a key player who is very stable in reception and she suddenly didn’t get her act together anymore in reception and shortly after that the same thing happened to the others.” Boss and Kleinert (2015) reported an analogical phenomenon, when they observed how balancing performance of one team member had a contagious effect on the other one. While their study was only examining teams consisting of two people, the current study is the first to discover decreased performance contagion as a cause of team collapse in teams consisting of more than two members.

Some players also found *cautious play* to be present within their team during a collapse as a result of insecurity and anxiety to fail. Athlete 4 (volleyball) said: “The setter doesn’t dare to play the right pass, the more difficult long way anymore, but focuses on simple passes, but then you can’t make the game complex anymore and it’s easy for the opponent to read the game. And that makes it harder for the attackers, which is why the error rate increases even more and that continues.” Wallace et al. (2005) found that pressure leads to higher levels of cautiousness in individual athletes in team sports. The level of cautiousness depends on the degree to which they try to avoid failure rather than to aim for success. They further argue that this failure avoidance behavior is closely related to negative performance outcomes. The authors also assume that cautiousness in team sports leads to athletes passing the ball to teammates more often instead of taking chances themselves. This behavior appears to be closely related to the cognitive outcome of lacking accountability that has been found to maintain team collapse in this study and the avoidance behavior that goes along with it. Cautious behavior in team sports therefore seems to be related to insecurity and anxiety to fail and to be characterized by athletes playing a more cautious style to avoid further mistakes.

Contrary to cautious play, athletes described how team collapse and the pressure created a *hectic rush* and made the players rush their actions in order to overcome the collapse and score. Athlete 6 (volleyball) described how a hectic rush, in order to end the collapse, made scoring even harder: “It’s the task of the setter to make the game calm but due to the hectic rush, setting became imprecise and due to that it was more difficult to make a point. It’s a vicious circle.” Apitzsch’s (2009) participants also mentioned that throws within the handball team were taken too quickly and from unfamiliar positions, what Apitzsch (2009) refers to as “making wrong decisions.” We assume though that a hectic rush was what caused those wrong decisions and quick throws in the first place.

Players further found that *limited communication* was important during the team collapse situation and prohibited the team’s recovery from the collapse. Athlete 2 (volleyball) explained: “We usually communicate a lot on the court; you realize the collapse when it gets quiet. No one calls for the ball anymore.” Some players even indicated that others pulled them down by communicating less after failure, like Athlete 7 (volleyball): “There was a lack of communication on the court. Two, three players had a down and pulled the others down, too. We should have talked to each other more to get out of this again.” While Apitzsch (2009) reported communication in the handball team to become particularly negative, participants in this study indicated that, independent from its valence, communication decreased significantly during a collapse, causing a loss of coordination and structure within the team. McEwan and Beauchamp (2014) argue that communication within the team is one of the most important teamwork behaviors that regulates team performance. They further explain that sharing information through communication allows for “moment-to-moment adjustments” in the team, such as tactical changes, that support a good performance. Contrary to this, limited communication seems to cause a performance decrease, which may be due to the inability of the team to adjust their tactics without communicating. Morgan et al. (2013, 2017) in this context argue that working communication channels foster team resilience when encountering stressors and should be facilitated in teams to foster resilience in high-pressure situations.

Athletes said that *blaming others* after failure also played a role during team collapse and fostered maintenance of the collapse. Athlete 10 (basketball), for example, admitted to show this behavior himself due to his increased nervousness: “[In team collapse situations], I become extremely nervous and I’m someone who doesn’t try to find the failures in his own play, but in others’ [play] instead.” This negative handling of each other’s failures within critical events seems to foster maintenance of team collapse. Jones and Harwood (2008) found negative criticism of team members to cause negative psychological momentum in sport teams. It appears that negative handling of previous mistakes can also cause maintenance of collective collapse. Morgan et al. (2013, 2015), similarly, argue that a no blame culture is very important for a team when experiencing failure.

## General Discussion

The main goals of this study were to gain an understanding of athletes’ perspectives on causes of collective team collapse and to define team collapse in contrast to negative momentum. The definition that developed from the interviews was: “We define collective team collapse as a sudden, collective, and extreme underperformance of a team within a competition, which is triggered by a critical situation that interferes with the team’s interplay, a loss of control of the game, and ultimately the inability of the team to regain their previous performance level within the game.” Our results show that the collectivity of the collapse manifests itself in the transfer of negative affect, cognition, and behavior within the team. These transfer processes within the team illustrate that collective team collapse is more than merely the sum of individual choking of multiple players at the same time. Our definition adds two valuable new aspects compared to Apitzsch’s (2006) previous definition: First, team collapse is collective and thus involves the whole team, and second, the team is unable to recover from it within the game. Future research in the area of team collapse to question or support this new definition is warranted.

Researchers have examined similar constructs to collective team collapse, such as the choking of individuals in a team setting (Hill and Shaw, 2013); however, this previous research mainly focused on individual factors causing individual underperformance in single or team sports. To date, group factors causing team collapse have not attracted sufficient empirical examination in previous studies, although the examination of team collapse on a team level is important to gain an understanding of group-related causes of this phenomenon. We therefore examined the causes, processes, and maintenance of team collapse as experienced by 10 team sport athletes to better understand the group-related processes as well as provide increased conceptual clarity and potential differentiation to negative momentum.

Regarding social or group-related causes of team collapse, unforced error accumulation, key player collapse, emotional contagion, decreased performance contagion, lack of accountability, limited communication, and blaming other players for mistakes were identified as social factors that cause and notably maintain team collapse in various situations. These factors differed from individual factors influencing team collapse (e.g., insecurity or cautious play), as they necessarily involved an interaction with other players. While insecurity, cautious play, or anger could also be expressed by an individual as an individual reaction to a bad game situation, social factors, such as a lack of accountability or blaming others for mistakes, are directly dependent on and necessarily involve the interaction of individuals on the court.

Results also showed that factors causing team collapse seemed to be interconnected within a temporal process that leads to team collapse and maintenance of this collapse. Jones and Harwood (2008), similarly, suggest a division between triggers and outcomes of psychological momentum but do not further distinguish between triggers and antecedents or between various outcomes of psychological momentum. The Multidimensional Model of Momentum (Taylor and Demick, 1994) proposes a precipitating event causing individual subjective changes in cognition, affect, and physiology depending on the individual experiences of the event. These changes are further believed to cause a change in behavior, performance, and subsequent outcome. Again, antecedents of the precipitating event are not included in Taylor and Demick’s (1994) model and underperformance is explained as an outcome of momentum, while it has been found to induce team collapse in this study.

The model proposed by Apitzsch (2009) divides causes of team collapse into causes before and during the match and makes further attempts to explain causal relations between emotional, behavioral, and cognitive factors influencing collective team collapse during a match. Apitzsch (2009) suggests a tentative relation of cognition causing emotion and emotion causing behavior before the start of a match and emotion causing cognition and cognition causing behavior after the occurrence of a team collapse event. Results of the current study partly support these suggestions (e.g., anxiety of failure causing a lack of accountability, which then causes cautious behavior during the game). Nevertheless, each interviewed athlete indicated his or her own tentative relation between affective, cognitive, and behavioral outcomes that maintain collective collapse. The reason for these discrepancies in athletes’ descriptions may be that they had experienced various collapse situations within different teams and different types of sport, whereas Apitzsch (2009) reports a specific team collapse event experienced by a single handball team. We therefore suggest to divide causes of collective team collapse into antecedents, critical events, and consequences as a chronological sequence as illustrated in Figure 1. We do not propose to include further directions of relations between affective, cognitive, and behavioral outcomes at this stage of research, since they have not yet been investigated sufficiently.

It is noticeable, although athletes from interactive ball team sports were included in the study in order to identify overall causes of team collapse relevant in a variety of team sports, that participants named a wide range of causes of collective team collapse. This variety of antecedents, critical events, and outcomes maintaining team collapse on the one hand illustrates the complexity of the phenomenon and the many factors that can, through combined appearance, trigger a team collapse. On the other hand, this variety may be an indicator for causes being of varying importance in evoking a team collapse depending on the type of team sport. Future research could therefore further investigate differences in causes of collective team collapse in different types of team sport.

As mentioned earlier, existing research does not sufficiently distinguish between the terms negative momentum and collective team collapse. Researchers have argued that individual athletes who choke differ from those who underperform (Hill and Shaw, 2013; Mesagno and Hill, 2013); specifically athletes who choke experience more intense emotions that they cannot self-regulate to recover their performance. Similarly, athletes in this study described that especially negative affect, such as anger and anxiety, was present, and transferred within the team during collective team collapse (i.e., negative emotional contagion) and prohibited the team from returning to a regular performance. Therefore, we consider it important to distinguish conceptually between negative momentum and team collapse. The “sudden, collective, and extreme underperformance of a team” and “the inability of the team to regain their previous performance level within the game” are integral elements of our definition of collective team collapse. According to this definition and our findings, collective team collapse seems to constitute a more extreme form of negative momentum (Cotterill, 2012), often with limited opportunity of returning to previous levels of performance within the game, while negative momentum can shift between teams (Adler and Adler, 1978). In other words, collective team collapse can be seen as a chronic and fatal underperformance of a team, while negative momentum is a temporary phase that can be overcome. Future research needs to clarify if and to what extent collective team collapse can grow out of negative momentum and what possible buffering factors are that allow a team to prevent the collapse when experiencing negative momentum.

### Limitations and Future Research

There are some limitations to the study. First, the interviews were conducted by seven different interviewers due to organizational settings of the study. Although one can argue that several interviewers are less subjective than one, this procedure does not pursue a strict interpretation of grounded theory, since themes developed during initial interviews may not shape later interviews in the same way as if conducted by the same interviewer. Besides that, the interview length of 20–40 min, due to the limited availability of professional athletes, has to be acknowledged as a further limitation. It cannot be ruled out that longer interviews may have created additional information. Another factor to be mentioned is that athletes may have been influenced in defining team collapse by the short colloquial description of the phenomenon that they received prior to the interview in order to make sure they knew what kind of phenomenon we were addressing. Moreover, the study only included athletes from elite sport levels. We are aware that team collapse situations can occur in amateur sports as well, which is why they should be included in future research. Another point to be raised is that the current study applied only one method to assess athletes’ perceptions in a single setting, using only one theoretical pathway to interpret data. Future studies should include multi-modal methods to capture participants’ perceptions across different settings and use more than one theoretical interpretation of the data. Furthermore, the study only revealed insights into athletes’ inner perceptions of antecedents, critical events, and outcomes as causes and maintaining factors of team collapse. In order to get a more global view of the phenomenon, outer perceptions of observers (e.g., coaches and sport psychologists) of team collapse should be considered as well. We suggest for future studies to include coaching staff’s perceptions of causes of team collapse within specific team sport settings. Future research should also engage in investigating the relationship between affective, cognitive, and behavioral consequences of critical team collapse events to gain a better understanding of possible starting points for future interventions. Besides that, sport-specific triggers of collective team collapse could be investigated.

The findings of the current study allow for the derivation of several applied implications for sport teams to prevent a team collapse, which also need to be investigated and tested for effectiveness in future research. Such applied implications could for example include the prevention of antecedents, which tend to increase the probability of a team to suffer from collective team collapse. Especially poor preparation or physical exhaustion from practices before the game could be prohibited by coaches and players. Furthermore, coaches and sport psychologists could help the team to react differently to a critical team collapse situation. Emotional regulation strategies have been shown to help prevent negative emotion and improve team performance (e.g., Tamminen and Crocker, 2013). Another suggestion is to coach key players or players with a leadership role in the team to keep the communication level up after a critical event and to communicate a mutual strategy to the players to prevent a decrease in communication and to foster team cohesion. In relation to blaming others for failure, a culture of no blame is associated with team resilience (Morgan et al., 2013) and could be established. Further ideas to make a team more resilient to collective collapse are the inclusion of simulation trainings and resilience trainings into practice on a regular basis. As mentioned before, the effectiveness of such applied interventions needs to be investigated in future studies prior to implementation.

## Conclusion

In this study, we explored athletes’ perceptions of causes of collective sport team collapse in various team sport disciplines. Major causes included emotional contagion, decreased performance contagion, lack of accountability, limited communication, and blaming other players for mistakes. These causes appeared to be interconnected in a temporal order consisting of antecedents, critical events, and outcomes that foster a maintenance of the team collapse. Based on these findings, we proposed a process model of causes of collective sport team collapse and a definition of team collapse to distinguish it from the related concept of negative momentum. The results provide first insights into causes of collective team collapse in a variety of sports and sport teams and found a basis for understanding what team collapse is and how it divides from other individual or group phenomena related to performance decreases.

## Author Contributions

The original research is part of the Ph.D. thesis of VW, supervised by JB. VW, ZZ, CM, and JB contributed to the conception and design of the study. ZZ and VW performed data collection and analysis. VW wrote the first draft of the manuscript. CM and JB wrote sections of the manuscript. All authors contributed to manuscript revision, read, and approved the submitted version.

## Conflict of Interest Statement

The authors declare that the research was conducted in the absence of any commercial or financial relationships that could be construed as a potential conflict of interest.
